# EEG study on implicit beliefs regarding sexuality: Psychophysiological measures in relation to self-report measures

**DOI:** 10.3389/fpsyg.2022.930863

**Published:** 2022-09-27

**Authors:** Robin van der Linde, Geert van Boxtel, Erik Masthoff, Stefan Bogaerts

**Affiliations:** ^1^Department of Developmental Psychology, Tilburg School of Social and Behavioral Sciences, Tilburg University, Tilburg, Netherlands; ^2^Graduate School of Behavioral and Social Sciences, University of Groningen, Groningen, Netherlands; ^3^Department of Cognitive Neuropsychology, Tilburg School of Social and Behavioral Sciences, Tilburg University, Tilburg, Netherlands; ^4^Fivoor Science and Treatment Innovation, Rotterdam, Netherlands

**Keywords:** sexual offenders, implicit beliefs, QITSO, psychophysiological profile, N200, P300, heart rate variability, resting-state activity

## Abstract

In this exploratory, correlational study, several psychophysiological measures were assessed and the relation between these measures and an experimental self-report questionnaire to measure the seven implicit beliefs of sexual offenders (the Questionnaire for Implicit Theories of Sexual Offenders (QITSO)) was established in a sample of Dutch participants recruited from the healthy population (*N* = 28) using correlational analyses. After analyzing task performance, electroencephalogram (EEG) data and electrocardiogram (ECG) data, the psychophysiological variables were correlated with the experimental QITSO subscales. The subscale “children as sexual beings” correlated positively with the P300 amplitude at electrode Pz. The subscale “women are unknowable” correlated positively with resting-state beta activity during eyes closed and eyes open, and with alpha activity during eyes open. Additionally, the subscale “entitlement’ correlated positively with low frequency heart rate variability power during eyes closed and eyes open, and with high frequency power during eyes closed. This study is a first exploratory step towards establishing a psychophysiological profile underlying the self-report questionnaire QITSO.

## Introduction

Sex offenders make use of cognitive distortions to justify their sexual offending behavior. Ward and Keenan (1999) proposed that these cognitive distortions arise from a set of core schemas held by the offender. According to Bem (1981), a schema can be described as a cognitive structure of learned associations, which directs attention, informs perceptions, and provides a shortcut for interpreting incoming stimuli (Fiske and Taylor, 1984; Tuente et al., 2019). Ward and Keenan (1999) suggest that these schemas are in fact implicit beliefs that the offender has about the world, for example, to explain other people’s actions and make predictions about the self and the world. These beliefs can be offense related, as they support, reinforce, or excuse offending behavior by, for example, diminishing negative outcomes or blaming the victim (Paquette and Cortoni, 2020). Ward and Keenan (1999) identified five implicit beliefs that describe the thought processes of sex offenders. These are children as sexual objects, entitlement, dangerous world, uncontrollability, and nature of harm.

The first implicit belief, children as sexual objects, conceptualizes children as beings with their own beliefs and desires and describes the thought process of child molesters. It describes the belief that humans, including children, are sexual beings and could enjoy and desire sexual activities (Bartels and Merdian, 2015). This implicit belief can lead to falsely interpreting a child’s daily behavior as a sign of sexual intentions and desires, for example, hugging the offender. This implicit belief can generate offense-related distorted statements, such as “the child seduced me,” to justify the committed offense (Ward and Keenan, 1999).

The second implicit belief, entitlement, is based on the core idea that some people are superior and that their wishes and needs are more important than those of others (Beech et al., 2009). For example, men are seen as more powerful than women and children. Therefore, men have the right to have their sexual needs met when and with whomever they want. This belief mainly refers to the thought process of rapists (Polaschek and Ward, 2002) and results in offense-related cognitive distortions, such as “People do what I ask them and that includes sex” (Ward and Keenan, 1999).

The third, dangerous world, is based on the belief that the world is a dangerous place where people pursue their own interests and needs by behaving in an abusive way (Bogaerts et al., 2004; Ildeniz and Ciardha, 2018). There are two variants of this implicit belief regarding rapists and child molesters. In the first sub-belief, the individual feels the need to dominate, punish, or control others, which can manifest itself in punishing those who supposedly harm the offender. This can lead to offense-related distortions, such as “I had to teach her a lesson” (Ildeniz and Ciardha, 2018). In the second sub-belief, adults are seen as unreliable, while children are seen as trustworthy. Thus, children are seen as loving and capable of giving love to the offender. The expectation here is that children are caring and able to fulfill the offender’s sexual needs. This results in cognitive distortions, such as “Children can give adults more acceptance and love than other adults” (Ward and Keenan, 1999).

The fourth implicit belief, uncontrollability, assumes that, in the context of sexual abuse, the male sex drive is uncontrollable. This belief states that there are uncontrollable deviant sexual preferences that leave the offender with no free choice and can only act on the sexual drive and thus cannot be held responsible for their abusive behavior (Bartels and Merdian, 2015). This implicit belief can lead to cognitive distortions, such as “I cannot control myself, so I’m not responsible” (Ward and Keenan, 1999).

The fifth, nature of harm, applies mainly to child molesters and is based on two assumptions. The first is that there are degrees of harm associated with sexual abuse, ranging from little or no harm to extreme harm. The probability of adverse consequences depends, for example, on the amount of force used by the offender, the victim’s awareness, and the social meaning of the abuse (Bartels and Merdian, 2015). Thus, since the victim could have been harmed more and the offender chose not to do this, he considers himself as someone who has taken care of the victim’s well-being and should not be judged too harshly. The second assumption is that sexual activities are beneficial and unlikely to harm anyone. The distress is not caused by the act itself, but by the society’s reaction to it (Drake et al., 2001). Together, these two assumptions lead to the judgment that children are unlikely to be harmed by the sexual experience, and that possible harm is the result of additional violence used by the offender, or of society’s response. This implicit belief results in offense-related cognitive distortions, such as “She is too young to remember this or know what I am doing,” or “The only way I could harm a child when having sex with her is to use physical force to get what I want” (Ward and Keenan, 1999; Höing et al., 2017).

In addition to these five implicit beliefs identified by Ward and Keenan (1999), there are two more implicit beliefs that can generate cognitive distortions that may be present in the thinking of sex offenders, and rapists as identified by Polaschek and Ward (2002). The first implicit belief is that women are unknowable. It states that women are naturally different from men and men cannot understand these differences. Believing that women are different by nature facilitates harming women because it is easier to harm someone who is seen as very different (Polaschek and Ward, 2002).

The second is that women are sexual objects who constantly desire sex and want to meet the sexual needs of men under all circumstances, even if it is coerced. This can lead to misinterpretations of their behavior as being sexual, for example, wearing a dress or being nice to a man can be seen as indicative of sexual interest (Polaschek and Ward, 2002). In conclusion, there are seven implicit beliefs, three of which are seen as antisocial beliefs, namely uncontrollability, dangerous world, and entitlement. These implicit beliefs influence not only the cognitive distortions of sex offenders, but also attention, social information processing and overt behavior (Drake et al., 2001; Tuente et al., 2019).

Various questionnaires have been developed to measure each of these seven implicit beliefs. The Molest Scale (Bumby, 1996), the Child Molester Scale (Cann et al., 1995), and the Offenses Against Children Scale (Lindsay et al., 2007), for example are used to measure implicit beliefs specifically of child molesters, such as children as sexual beings or nature of harm. Several questionnaires have also been developed to measure implicit beliefs that apply specifically to rapists. These include, for example, the Hostility Towards Women Scale (Check, 1985), the Attitudes Toward Women Scale (Spence et al., 1973), and Bumby’s RAPE Scale (Bumby, 1996). These measure implicit beliefs such as women are sexual objects. According to Gannon et al. (2008), some problems exist in the questionnaires currently used to measure implicit beliefs. These questionnaires often fail to accurately measure the antisocial implicit beliefs uncontrollability, entitlement, and dangerous world. Therefore, the experimental version of the QITSO was developed by Noteborn et al. (2020) to measure the seven implicit beliefs described previously, including the antisocial beliefs. The QITSO was designed to accurately measure each of the seven implicit beliefs combined in one instrument and it is inspired by several existing questionnaires.

However, when it comes to self-reports, there are several limitations (Paulhus and Vazire, 2007). When using self-report questionnaires, test scores are influenced by non-test-relevant characteristics of the respondents (Crowne and Marlowe, 1960). Self-reports are prone to deception and self-presentationfval strategies (De Houwer, 2002). An example of this is social desirability bias, which is defined as the desire to make a favorable impression on others (Tan and Grace, 2008). This social desirability bias poses a serious threat to the validity of a questionnaire, especially if there is a large social value placed on the items. Due to concerns about self-representation, participants have the desire to underreport socially undesirable thoughts and overreport socially desirable ones (Krumpal, 2013). Especially in the context of sensitive questions, answers are often distorted by the social desirability bias, and this can distort the information obtained from self-reports (Jo, 2000).

In 1964, Crowne and Marlowe (1964) developed the Marlowe–Crowne Social Desirability Scale to address the issue of social desirability. However, the scale does not accurately measure how much each of the sensitive items is affected by this bias. It tells you whether someone is answering in a socially desirable way, but it does not provide you with a method for correcting the answers given to those sensitive items (Jo, 2000). Therefore, researchers sought another method to control for biases in self-report measures. Several studies have used the implicit association test (IAT) to investigate the problematic cognitions of sex offenders more indirectly (Nunes et al., 2007; Brown et al., 2009; Kanters et al., 2016). The IAT is used to test implicit associations and beliefs (Dawson et al., 2009) and the idea behind the IAT is that individuals should respond faster when two concepts (stimuli) are closely linked in memory and respond slower when two concepts are less closely associated. For example, a study by Milhailides et al. (2004) found that child molesters responded faster to word pairs, such as child and lust compared to a non-offending control group. However, studies assessing implicit beliefs with the IAT have so far focused only on clinical samples of sex offenders. To our knowledge, there is no research on implicit beliefs related to sexually deviant behavior in a community population. Therefore, the most important studies conducted on sex offenders are discussed and the results cannot be extrapolated to the general population.

Research on implicit beliefs in sex offenders so far has focused mainly on child molesters, and especially on the implicit belief that children are sexual objects. For example, Nunes et al. (2007) investigated sexual attractiveness in a group of 27 male child molesters and 29 male non-sex offenders, and found that child molesters view children as more sexually attractive compared to non-sex offenders. This implicit association between children and sex was also confirmed in the study by Brown et al. (2009). However, until now, only one study has used a measure other than self-report to assess another implicit belief, namely dangerous world. Kanters et al. (2016), assessed whether child molesters are sexually attracted to submissiveness. Because child molesters may believe that the world is a dangerous place, they might try to achieve dominance and be attracted to submissiveness. The results of this study indicated that child molesters have a stronger sexual preference for submissiveness than rapists, but no differences were found between child molesters and non-sex offenders. So far, only two of the seven implicit beliefs have been assessed by a measure other than self-report.

Our study sets out to find additional measures to complement the information about the seven implicit beliefs that is obtainable with a self-report questionnaire. In forensic psychiatry, there is a growing interest in brain research to find biological correlates or markers of deviant behavior. The assumption is that this new information can supplement the information that can be obtained using more traditional psychological methods, such as self-report questionnaires (Firth et al., 2018). The goal of this study is to investigate whether the measure of implicit beliefs can be complemented with the use of psychophysiological data, as sexual behavior is influenced by physiological mechanisms. For example, sexual stimulation originates in the brain and is triggered by input from the senses (Levin and Riley, 2007), and neurotransmitters are involved in the control of sexuality (Cour et al., 2013).

Psychophysiological studies have generally shown that cognitive impairment is related to sexually deviant thoughts and behavior. Following the classical neurobiological hypothesis of sexual deviance, there is a prominent role of basal fronto-temporal anomalies and fronto-temporal-limbic dysfunction, especially in the left hemisphere (Joyal et al., 2007, 2014). However, these anomalies and their behavioral manifestations are not specific and do not apply only to sex offenders. These anomalies appear to be associated with a wide array of disorders ranging from conduct disorders to schizophrenia and sexual offending (Joyal et al., 2014). Another limitation is that past studies mainly focused on different measures in various patient groups to assess a wide range of cognitive capacities, which could explain the divergent and non-specific results obtained in previous research (Joyal et al., 2007; Morais et al., 2015).

It is currently impossible to conclude whether specific psychophysiological response patterns are related to sexually deviant thoughts and behavior. Measures targeting more specific psychophysiological response dimensions rather than broad cognitive categories, such as executive functioning, may help identify different risk factors associated with deviant sexual thoughts and, possibly, behavior (Joyal et al., 2014). To establish whether specific psychophysiological response patterns are related to sexually deviant beliefs and behavior, the present study focuses on two types of psychophysiological measures, electroencephalogram (EEG) to measure the activity of the central nervous system and electrocardiogram (ECG) to measure the activity of the autonomic nervous system. Tasks of inhibition and cognitive processing are included and will be measured by event-related potentials (ERPs) in a non-clinical population (Sur and Sinha, 2009). ERPs are small electrical potentials generated in brain structures and ERPs occur in response to specific internal or external stimuli. ERPs represented EEG changes that can provide information about a wide range of sensory, cognitive, affective, and motor processes. ERPs are the summed activity of electrical potentials that are produced when a large number of similarly oriented neurons fire in synchrony (Sur and Sinha, 2009). ERPs can be recorded noninvasively and are therefore a common tool used in research.

Response inhibition is defined as the ability to suppress a prepared but not yet initiated action (Verbruggen and Logan, 2009) and deficits in response inhibition have been investigated in sexual offenders. However, results have not been congruent. Schiffer and Vonlaufen (2011) investigated the difference in executive functioning between pedophilic and non-pedophilic child molesters, non-sex offenders and a healthy control group. The results of this study showed that, compared to healthy controls and non-sex offenders, pedophilic and nonpedophilic child molesters showed deficits in response inhibition. The second study investigated differences in impulsivity between rapists, child abusers and non-sex offenders (Perley-Robertson et al., 2016). However, no significant differences were found between the three groups. A way of measuring response inhibition is with the Go/NoGo task, where participants must respond to one stimulus and refrain from responding to another stimulus. The associated ERP to these cues is called the N200, and this NoGo N200 has been linked to inhibition processes (Rosburg et al., 2018). Previous studies have also used the Stroop task to assess response inhibition in sex offenders (Adjorlolo and Egbenya, 2016). However, the Stroop paradigm makes only partial use of the concept of response inhibition, as it mainly tests the ability to pay attention to goal-relevant stimuli, and to ignore distracting stimuli, related to the ability to suppress interference.

Another component of executive functioning is cognitive processing, measured by the P300. The P300 reflects processes that are involved in stimulus evaluation or categorization and are related to novelty and information processing (Picton, 1992). Regarding sexuality, there is little research on cognitive processing as measured by the P300. The P300 component has been studied in relation to sexual desire levels. In a study conducted by Steele et al. (2013), the P300 component was measured in relation to pleasant-sexual, unpleasant, and neutral stimuli in a normal population. Results indicated that P300 mean amplitude in the neutral condition was less positive compared to both the unpleasant and pleasant-sexual conditions. Also, the P300 mean amplitude for the pleasant-sexual condition was more positive than the unpleasant and neutral conditions. However, to the best of our knowledge, whether there are fundamental underlying deficits in cognitive processing associated with sexually deviant beliefs, has not been investigated. Deficits in cognitive processing have been studied extensively in relation to a wide range of other conditions, such as externalizing problems (Patrick et al., 2006) and psychopathy (Kiehl et al., 1999). Furthermore, it is well established that reduced amplitude of the P300 can play a role in disorders related to the externalizing spectrum, such as substance abuse and antisocial disorders, which act as risk factors for aggression.

In addition to the N200 and the P300, this study will also use EEG and ECG recordings measured during resting state. ECG measures indicative of heart rate variability (HRV) are included because deficits in affective state regulation may contribute to the offending process, as research has shown that sex offenders experience difficulties with emotion regulation (Ward et al., 1997; Howells et al., 2004). Previous research has indicated that lower levels of heart rate variability (HRV) were frequently observed in patients with a variety of disorders, such as mood and anxiety disorders (Garakani et al., 2009), suggesting a link between HRV and the ability to regulate affective and emotional states.

The aim of the present study is investigate the possibility of establishing a psychophysiological profile underlying implicit beliefs. It involves a combination of a behavioral measure (the QITSO score) and physiological measures (N200, P300, EEG recordings, and ECG recordings). The importance of this study is twofold. First, this study goes beyond assessing just sexual attraction, as was done with the IAT and in the P300 studies. This study investigates underlying psychophysiological differences on neutral stimuli in relation to the seven implicit beliefs as measured by the experimental version of the QITSO. Second, this study uses specific cognitive measures that go beyond the general conclusion that sex offenders have deficits in cognitive functioning. This study investigates in more detail where these specific differences occur. This study includes psychophysiological measures of the central nervous system (EEG) and the autonomic nervous system (ECG), which reflects both cognitive and affective processes. The study is being conducted in a community sample due to the explorative nature of the study and because there is currently no theoretical ground to conduct the study in a sample of sex offenders.

## Materials and methods

### Participants

The original sample consisted of 35 male subjects of Dutch nationality, of which seven could not be included due to artifacts or similar problems that made the data unusable, leaving a total of 28 participants, with a mean age of 24 years (*SD* = 8.978, range 17–56). Twenty-three participants were right-handed (82.14%) and five were left-handed (17.86%). Participants were partly recruited at Tilburg University and earned course credits by participating. Another way of recruiting was done through convenience sampling. Inclusion criteria were male gender and speaking Dutch and exclusion criteria were female gender and having a neurological disorder that might influence perception or cognition. Because the experimental version of the QITSO is a Dutch questionnaire, participants had to be proficient in the Dutch language. Participation was completely voluntary, and participants could withdraw at any time. Informed consent was obtained from all participants.

### Procedure

**Figure 1:**
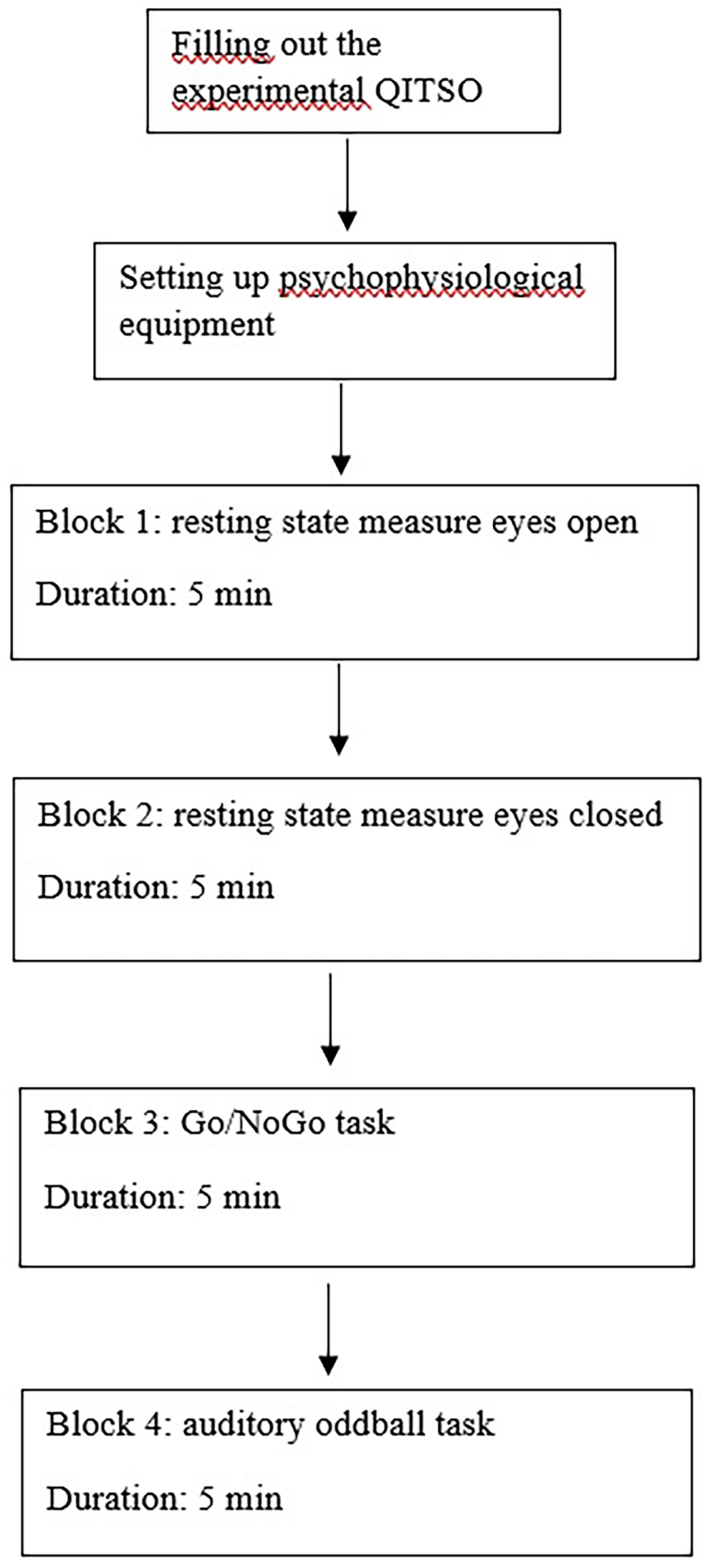
Graphical Presentation of the study.

The study took place at the Cognitive Neuropsychology lab at Tilburg University and each session lasted about 90 min. The test leader communicated with the participants according to a protocol to avoid bias in the interaction process. The procedure is summarized in Figure 1. Participants were first instructed to complete the QITSO, as a measure of the seven implicit beliefs. Then, participants were prepared for the psychophysiological recordings and took part in the experiment, which consisted of four blocks of about 5 min each. The first block was a resting state measure, where participants were instructed to look at the computer screen with their eyes open for 5 min, and in the second block they were asked to sit still for 5 min with their eyes closed. In the third block, participants performed the Go/NoGo task, and the final block consisted of the auditory oddball task. Participants performed the tasks in a sound-attenuating cabin on a computer 1 meter away. The refresh rate was 100 Hz. Participants were instructed to press the response keys (“b” for the Go/NoGo task) with their preferred hand. After finishing both tasks, the test leader debriefed the participants and thanked them for their participation.

### Behavioral measures

#### QITSO

As discussed earlier, this study focusses on the seven implicit beliefs believed to be used by sex offenders to account for their deviant behavior, namely children as sexual objects, entitlement, dangerous world, uncontrollability, nature of harm, women are unknowable, and women are sexual objects. The experimental version of the QITSO (Noteborn et al., 2020) consists of 130 items that can be scored on a scale of 1 (completely disagree) to 5 (completely agree). The implicit belief, children as sexual objects, is measured with 20 items and an example item is “Some children are willing to have sexual contact with adults.” The implicit belief, entitlement, is measured with 12 items, for example “Women should obey the sexual needs of a man.” Dangerous world is measured with 22 items and an example item is “Children are more reliable and trustworthy than adults.” The implicit belief, uncontrollability, is measured with 24 items, including “In many cases, men do not plan their sexual offenses with children; it just happens.” The fifth implicit belief, nature of harm, includes 21 items such as “Sexual contact between a man and a child is not harmful (physical and/or emotional) for the child.” Women are unknowable is measured with nine items. An example is “Rape is often the result of a wrong interpretation of signals.” The last implicit belief, women are sexual objects, is measured with 22 items, such as “Women who often go out are mainly looking for sex.” The QITSO is currently still in development and there is no information on reliability and validity. The internal consistency of the QITSO in the current study, as indicated by Cronbach’s alpha was *α* = 0.84.

#### Go/NoGo task

The Go/NoGo task was implemented as a measure of response inhibition and the associated N200 ERP component. The participants viewed two types of stimuli presented at the center of a computer screen and they were instructed to press the keyboard key “b” with their preferred hand as quickly and accurately as possible when the Go-stimulus, a green square, appeared. When the NoGo-stimulus, a red square, appeared, they were told to withhold their response and wait for the next stimulus. All stimuli were 100 ms in duration and the interstimulus interval varied *via* a rectangular distribution between 1.5 and 2.5 s. The block consisted of 200 trials (50% Go-trials and 50% NoGo-trials). Reaction times to Go stimuli and accuracy of response inhibition were recorded.

#### Auditory oddball task

The auditory oddball task was implemented as a measure of cognitive processing and the associated P300 ERP component. The participants were presented with two types of auditory stimuli, one standard tone of 1,000 Hz, and one target tone of 2,000 Hz. Participants were asked to silently count the target stimuli and to ignore the standard tones to ensure focus of the participants. All stimuli were 100 ms in duration and the interstimulus interval varied randomly between 2 and 3 s. The block consisted of 200 trials (80% standard stimuli, 20% target stimuli). After the 200 tones, participants were asked to indicate how many times they heard the target tone of 2000 Hz.

#### Psychophysiological equipment

Nexus-32 EEG recording equipment was used to provide 24 unipolar channels of continuous EEG recording (Mind Media B.V. with Biotrace Software Herten, The Netherlands) with a sampling frequency of 256 Hz. The EEG was recorded from four midlines ((Fz, Cz, Pz, Oz) and 16 homologous scalp locations (Fp1, Fp2, F3, F4, F7, F8, C3, C4, T3, T4, T5, T6, P3, P4, O1, O2) using an electrode cap. All EEG signals were referenced to a signal from an electrode placed on the left mastoid (A1), and EEG was also recorded from an electrode placed on the right mastoid (A2) so that an average-mastoid reference could be used in the analyses. An anterior midline site electrode (AFz) served as the ground electrode. Following cap placement, a small amount of abrasive Electro-Gel (Nuprep) was placed into each of the scalp and mastoid electrodes. After pressing the gel into the electrode and creating good contact between the scalp and electrode, a small quantity of electrolytic conducting gel was inserted into each electrode. Signals from all EEG channels were recorded using the Biotrace Software. Eye movements were monitored by two pairs of electrodes. The first pair was placed in a straight line below and above the right eye to track vertical eye movements. To track the horizontal eye movement, te other pair was placed in a straight line from the left temple to the right temple. The eye movement records were used to correct the ERPs for electrical contamination of horizontal and vertical eye movement artifacts following Gratton et al. (1983).

The electrocardiogram (ECG) was recorded bipolarly from 10 mm Ag/AgCI electrodes using a V6 versus sternum lead, which maximizes the R-wave with respect to other ECG components, and is relatively insensitive to artifacts (Mulder, 1988). Nexus-32 EEG recording equipment was used to provide continuous ECG recording during the eyes closed and eyes open (Mind Media B.V. with BioTrace Software Herten, The Netherlands) with a sampling frequency of 2048 Hz.

#### Psychophysiological recordings during the Go/NoGo and the auditory oddball task

Data analysis of the EEG recordings during the two tasks was performed offline using the Brain Vision Analyzer (2.2.0). First, the frequency was lowered from 2048 to 256 Hz. The reference was recalculated off-line to the linked mastoids (A1 + A2)/2, and low-pass filtering at 30 Hz (48 dB/octave) was used. The EEG was segmented into epochs starting 200 ms prior to the stimulus until 1,000 ms after the stimulus, both in the Go/No-Go and in the oddball tasks. Epochs were excluded from further analysis when the minimum and maximum of the low-pass filtered potentials differed by more than 200 μV (after correction for eye movements and blinks). The mean voltage measured in the 200 ms interval before the stimulus was used for baseline correction.

#### Psychophysiological recordings during the resting state

Resting state data were recorded while the participants were sitting quietly in the experimental room; 5 min with their eyes open focused on the computer screen, and 5 min with their eyes closed. The EEG signals recorded during these intervals were segmented in periods of 4 s with 75% overlap. That is, the first period consisted of activating during the interval from zero to 3 s, the second interval from one to four, then from two to five, etc. The EEG activity in each segment and each electrode was corrected for eye movements and blinks, and checked for artifacts, in the same way as the ERP segments (see above). Then the Fourier transform was computed for each segment and the transformed segments were averaged to obtain a stable spectrum (Welch, 1967). Theta (4–8 Hz), Alpha (8–12 Hz), and Beta (12–30 Hz) activities were then computer from these spectra, separately for eyes closed (EC) and eyes open (EO). Theta waves were measured with the Fz electrode, Alpha waves with the Pz electrode and Beta waves with the Cz electrode. We then computed differences scores for the Theta, Alpha, and Beta waves comparing eyes closed to eyes open.

The method of Afonso et al. (1999) was used to detect R-peaks from the ECG. Interbeat intervals were then computed to quantify HRV parameters, after correction using the RHRV v4.2.5 package (Rodriguez-Linares and Simpson, 2019). The RHRV package was also used to calculate the following HRV variables: mean heart rate frequency (meanHR), the interval between the R peaks (meanRR), the standard deviation of the intervals between the R peaks reflecting global heart rate variability (SDNN), root-mean-square of the successive differences reflecting fast variation in heart rate (RMSSD), the power in the low-frequency band (LF; 0.04–0.15 Hz), the power in the high-frequency band (HF; 0.04–0.15 Hz), and the ratio between the LF and HF bands (LFHF) The power in the HRV frequency bands was log-10 transformed to make the distribution normal.

### Statistical analyses

In this study, IBM SPSS Statistics version 24 was used to run the analyses. First, it is tested what the task performance was on both the Go/NoGo and the auditory oddball task, and mean scores on each of the seven implicit beliefs of the QITSO were calculated. The task performance items for the Go/NoGo task (reaction time; number of correct responses; number of correct inhibitions) and the task performance on the auditory oddball task (number of indicated target tones) were correlated with the QITSO and its subscales.

The third step was analyzing the EEG data. The ERPs were estimated by scoring the most negative (N200) peak in the interval of 100–300 ms after presentation of the stimulus and positive (P300) peak in the interval of 250–400 ms after presentation of the stimulus. When these peaks were estimated, five samples were taken around these peaks, reaching a total of 43 ms, as estimation of the peak amplitude. A General Linear Model (GLM) repeated measures analysis was calculated (a given condition X anteriority X laterality). Both the anteriority and the laterality consisted of three levels. For the anteriority these levels were frontal, central, and parietal. For laterality, these levels were left hemisphere, midline, and right hemisphere. For both tasks this means that a 9 GLM repeated measures design was used, consisting of 2 (conditions) X 3 (anteriority) X 3 (laterality). For the Go/NoGo task the two conditions were the Go and the NoGo trials; for the auditory oddball task the two conditions were the standard and the target tones. The GLM repeated measures analysis was used to determine which electrodes showed the greatest difference between the two different conditions for each task. Literature showed that with the Go/NoGo task, the effect was supposed to take place at the frontal midline for the NoGo trials (Van Boxtel et al., 2001). For the auditory oddball task, the effect was supposed to take place at the parietal midline for the target tones (Linden, 2005). The two electrodes that best showed the N200 and P300 peaks were selected for further analyses. For these two electrodes, a difference score was calculated by subtracting the N200 for the Go trials from the N200 for the NoGo trials and the P300 for the standard tones from the P300 at Pz for the target tones. These difference scores will be correlated with the QITSO and its subscales.

Lastly, the various physiological items related to the resting state measure with eyes closed and eyes open were correlated with the QITSO and its subscales. For the EEG data, the Theta, Alpha, and Beta activity were used both for eyes closed (EC) and eyes open (EO). For the heart rate data, meanHR, meanRR, the SDNN, the RMSSD, the power in the LF band, the power in the HF band, and the LFHF during eyes closed (EC) and eyes open (EO) were used.

## Results

### Task performance

Task performance on the Go/NoGo task was fast and accurate. The proportion of correct responses on the Go trials and correct inhibitions on NoGo trails were both high. The mean reaction time was fast (231 ms, SD = 429.2); therefore the participants did not seem to delay their responses to increase the likelihood of withholding the response when a NoGo signal occurred. The percentage of correct responses was 99.86% and the percentage of correct inhibitions was 97.47%. These results show that using the Go/NoGo task, response inhibition could accurately be measured. Regarding the auditory oddball task, participants estimated the number of deviant tones to range from 32 to 40 (target = 40). The percentage of correct responses was 67.86%, with a mean of 39.143. This value is almost the number of target tones included in the task, meaning that participants have paid attention while listening to the tones, so cognitive processing could accurately be assessed.

### QITSO and task performance

Results in Table 1 show the scores on the QITSO and on the seven implicit beliefs. This table shows that the scores on the QITSO and on the seven subscales are all on the lower end. This was to be expected, as the sample used in the current study consisted of people from the general population and not a clinical sample of sex offenders. Correlations between the QITSO scores and the task performance items can be found in Table 2. There are no significant correlations between the QITSO total score and the scores on the subscales, and the task performance items for the Go/NoGo and auditory oddball tasks.

**Table 1:** QITSO (experimental version).

Scale	Number of items	Minimum score	Maximum score	Mean	Range of observed scores
Total score	130	130	650	326	288–383
Children as sexual beings	20	20	100	39.75	28–56
Entitlement	12	12	60	20.64	16–28
Dangerous world	22	22	110	52.57	43–72
Uncontrollability	24	24	120	72.86	60–95
Nature of harm	21	21	105	41.39	31–49
Women are unknowable	9	9	45	19	12–28
Women as sexual objects	22	22	110	43.43	35–59

**Table 2:** Correlations between the experimental QITSO and task performance on the Go/NoGo and auditory oddball task.

Variables	% correct responses Go trials^1^	% correct inhibitions NoGo trials^2^	Reaction time^3^	Indicated number of target tones^4^	Total score QITSO^5^	Children as sexual beings^6^	Entitlement^7^	Dangerous world^8^	Uncontrollability^9^	Nature of harm^10^	Women are unknowable^11^	Women as sexual objects^12^
1	1.000	˗0.006	˗0.217	0.814^*^	˗0.081	˗0.125	˗0.131	˗0.090	˗0.048	0.150	˗0.142	0.048
2		1.000	0.394^*^	˗0.052	˗0.034	˗0.120	0.009	0.068	˗0.119	0.218	˗0.043	0.052
3			1.000	˗0.395^*^	˗0.259	˗0.195	˗0.004	˗0.269	˗0.368	0.190	0.062	˗0.102
4				1.000	˗0.073	˗0.070	˗0.128	0.050	˗0.021	0.155	˗0.337	˗0.053
5					1.000	0.600^**^	0.345	0.737^**^	0.779^**^	0.325	0.501^**^	0.681^**^
6						1.000	0.219	0.284	0.325	0.158	˗0.024	0.070
7							1.000	0.174	0.208	0.021	0.026	0.105
8								1.000	0.450	0.039	0.398^*^	0.581^**^
9									1.000	0.166	0.356	0.453^*^
10										1.000	˗0.109	0.187
11											1.000	0.615^**^
12												1.000

** *p* < 0.01;

* *p* < 0.05.

### Go/No-Go task

**Figure 2:**
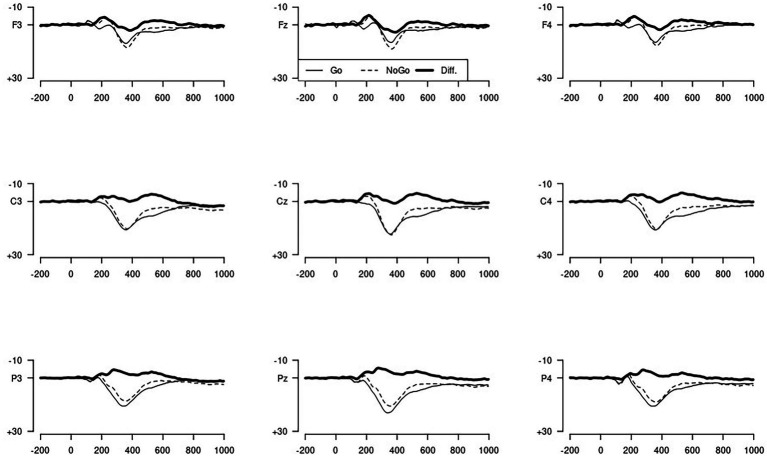
Overview of event-related potentials for Go and NoGo trials.

**Figure 3:**
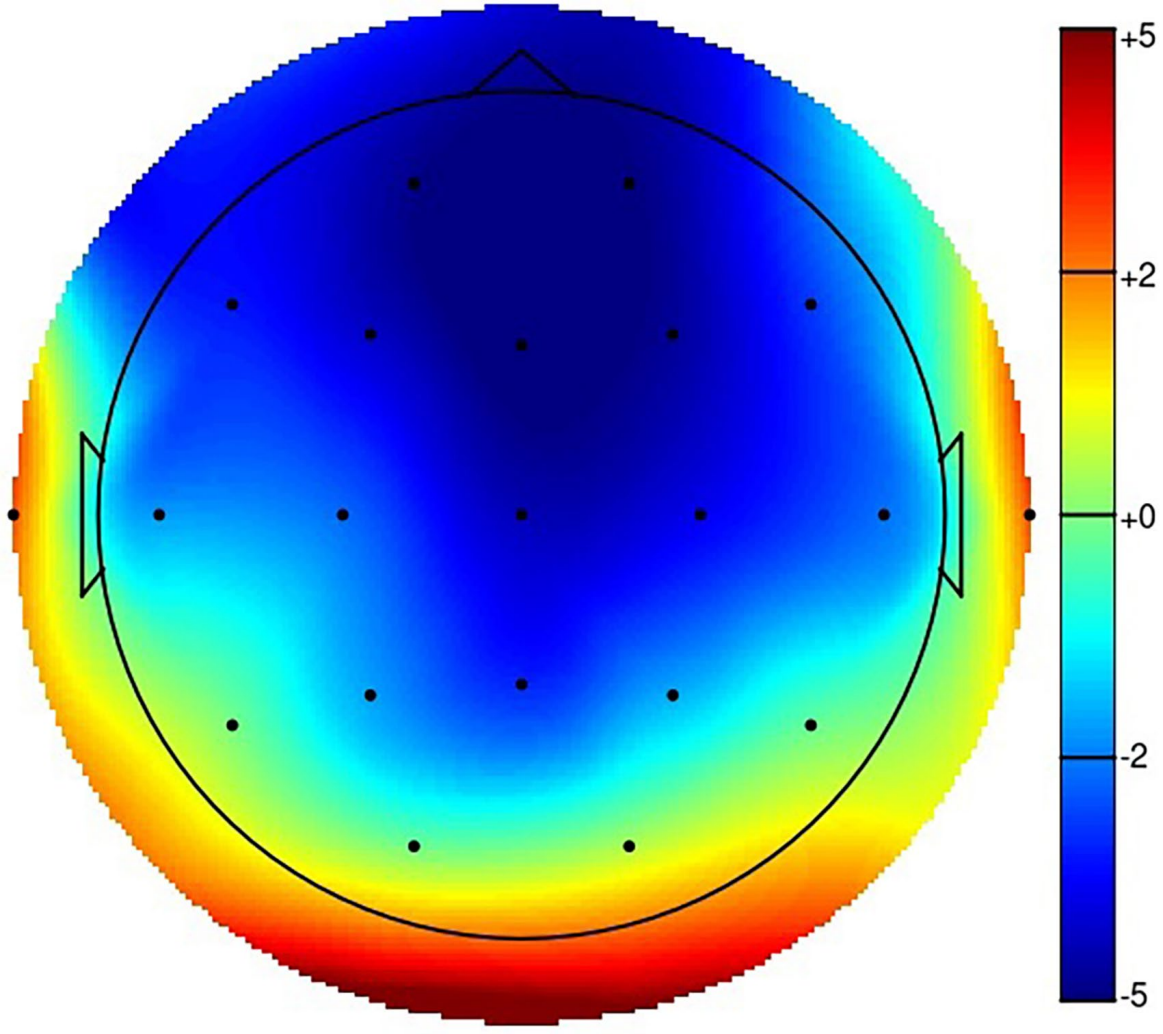
Topographical map of the N200. NoGo - Go, 218 ms.

The results of the N200 in the Go/NoGo task are shown in Table 3, and the corresponding waveforms are shown in Figure 2. Figure 3 represents a topographical map for the N200. The N200 was greater for the NoGo compared to the Go stimuli, and greater at frontal than at other electrode sides. There was no difference between the two hemispheres, but at frontal and central electrodes, the N200 was larger on the midline compared to the lateral sides. As can be seen from the graphs, the difference between the two conditions, or the N200 amplitude, is greatest for the Fz and Cz electrodes, which is as expected located at the frontal midline of the scalp. For the Fz electrode, the difference between the two conditions was calculated.

**Table 3:** Results of GLM repeated measures for the Go/NoGo task.

Source of variation (effect)	*F*	*df* (error *df*)	*η^2^*	*P*
Condition	30.927	1 (27)	0.534	<0.001
Anteriority	41.399	2 (26)	0.761	<0.001
Laterality	3.334	2 (26)	0.204	0.051
Condition * Anteriority	2.111	2 (26)	0.140	0.141
Condition * Laterality	7.447	2 (26)	0.364	0.003
Anteriority * Laterality	8.266	4 (24)	0.579	<0.001
Condition * Anteriority * Laterality	3.069	4 (24)	0.338	0.036

### Auditory oddball task

**Figure 4:**
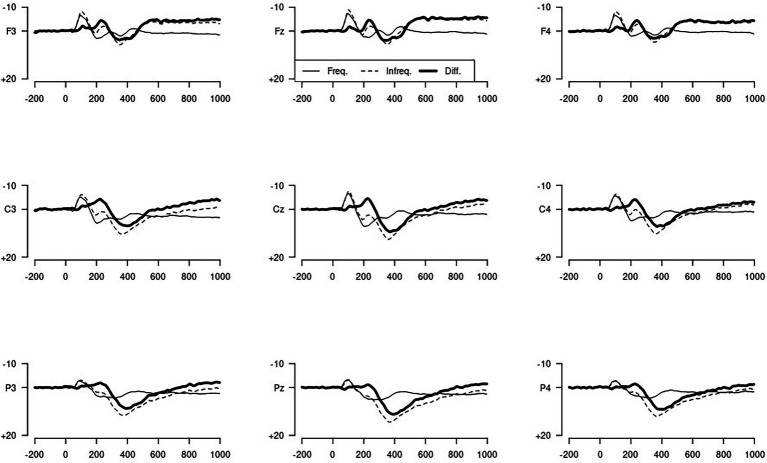
Overview of event-related potentials for Standard and target tones.

**Figure 5:**
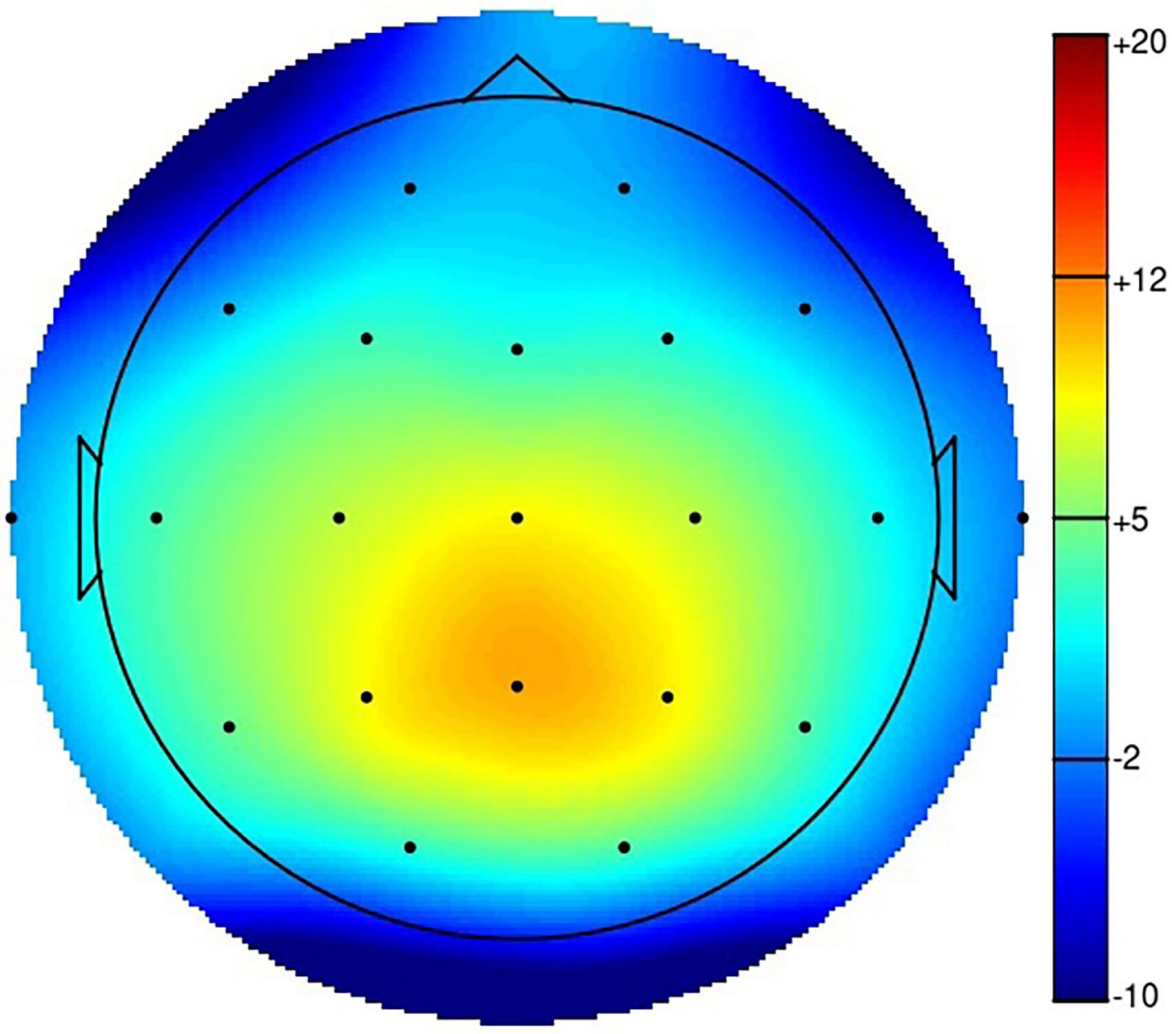
Topographical map of the P300. Infrequent - frequent, 400 ms.

The results of the P300 in the auditory oddball task are shown in Table 4, and the corresponding waveforms are shown in Figure 4. Figure 5 represents a topographical map for the P300. The P300 was greater for the target tones in comparison to the standard tones, and greater at parietal than at other electrode sides. There was no difference between the two hemispheres, but at parietal and central electrodes, the P300 was larger in the midline compared to the lateral sides. As can be seen from the graphs, the difference between the two conditions, or the P300 amplitude, is greatest for the Pz electrode, which is located at the parietal midline of the scalp, as expected. For this elecstrode, the difference between the two conditions was calculated.

**Table 4:** Results of GLM repeated measures for the auditory oddball task.

Source of variation (effect)	*F*	*df* (error *df*)	*η^2^*	*P*
Condition	26.669	1 (27)	0.497	<0.001
Anteriority	65.340	2 (26)	0.834	<0.001
Laterality	20.778	2 (26)	0.615	<0.001
Condition * Anteriority	27.544	2 (26)	0.679	<0.001
Condition * Laterality	5.697	2 (26)	0.305	0.009
Anteriority * Laterality	7.154	4 (24)	0.544	0.01
Condition * Anteriority * Laterality	2.761	4 (24)	0.315	0.051

### QITSO and the difference scores

Results of the correlations between the QITSO, its subscales, and the difference scores for the Go/NoGo and the auditory oddball task showed a small significant, positive correlation between the oddball task difference score and the subscale children as sexual beings *r* = 0.373, *p* = 0.050. No other significant correlations between the difference scores and the QITSO were found, as shown in Table 5.

**Table 5:** Correlations between the experimental QITSO and the difference scores for the Go/NoGo and the auditory oddball task.

Difference scores	Total score QITSO	Children as sexual beings	Entitlement	Dangerous world	Uncontrollability	Nature of harm	Women are unknowable	Women as sexual objects
N200(Fz)	˗0.146	0.099	0.019	˗0.229	˗0.026	˗0.075	˗0.098	˗0.266
P300(Pz)	0.364	0.373*	˗0.045	0.057	0.352	0.174	0.223	0.137

* *p* < 0.05.

### Resting state data

**Figure 6:**
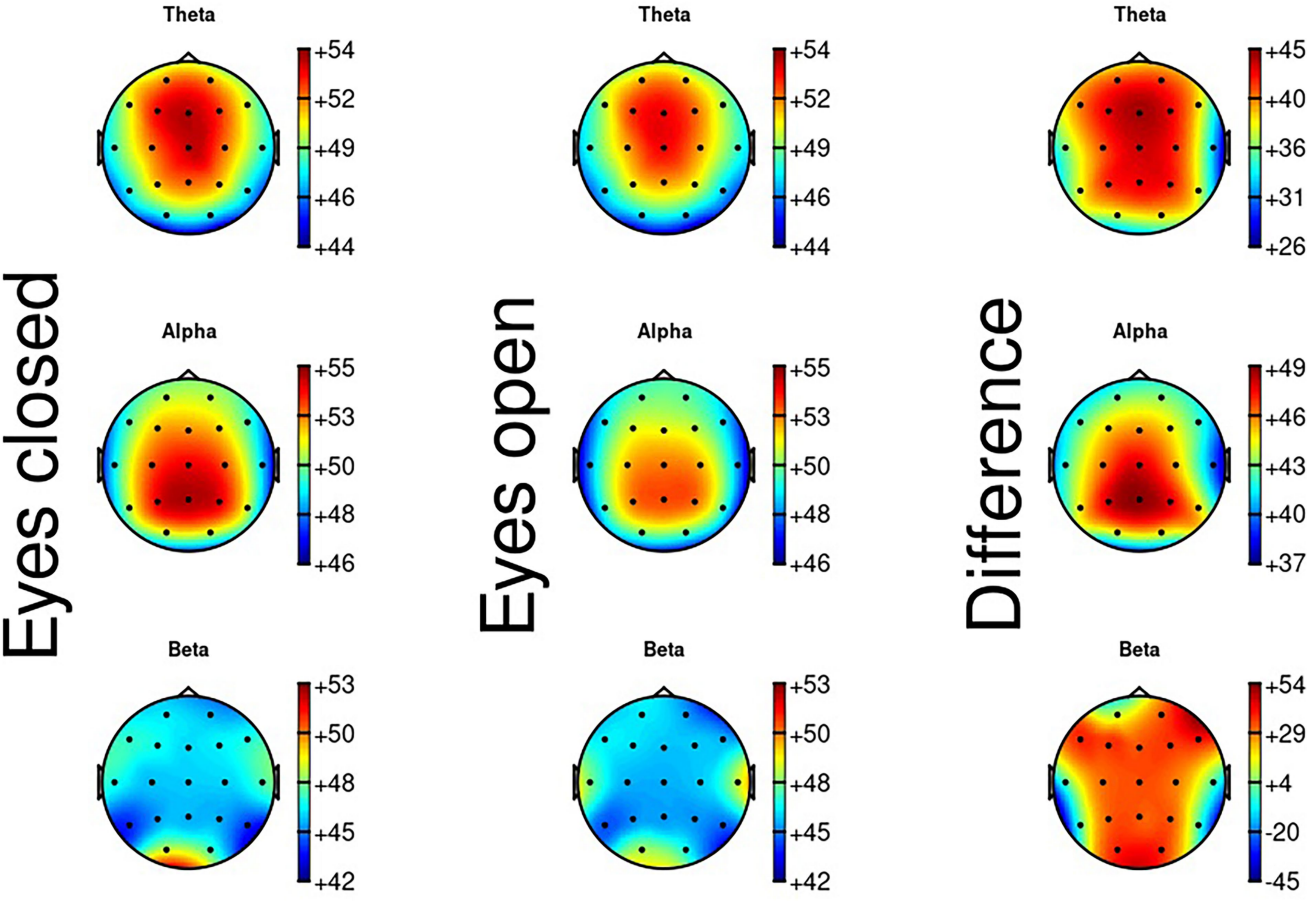
Topographical map of theta, alpha and beta activity during eyes closed, eyes open, and the difference between eyes closed and eyes open.

For the EEG eyes closed measures, the alpha peak was around 8–12 Hz, which is the value typically found in the literature (Murphy and Öngür, 2019). Figure 6 shows the topographical maps of the theta, alpha and beta waves for eyes closed, eyes open and the difference between eyes closed and eyes open. Regarding theta activity, there is a focus on the frontal midline of the scalp, which is where the theta activity normally occurs (Inanaga, 1998). Alpha activity mainly occurs around the Pz electrode, located at the parietal midline of the scalp, which is where alpha activity normally occurs (Kropotov, 2009). Regarding beta activity, this is normally most prominent at the midline of the scalp (Kropotov, 2009), but the map does not show this very clearly. For EEG eyes open, results were identical to results found in the EEG eyes closed measures.

For the ECG measures during the resting state, activity of the spectrum of the heart rate variability during eyes closed ranged from 0 to 0.4 Hz. There was more variation in heart rate variability during the eyes closed condition compared to the eyes open condition.

### QITSO and the EEG resting state

**Figure 7:**
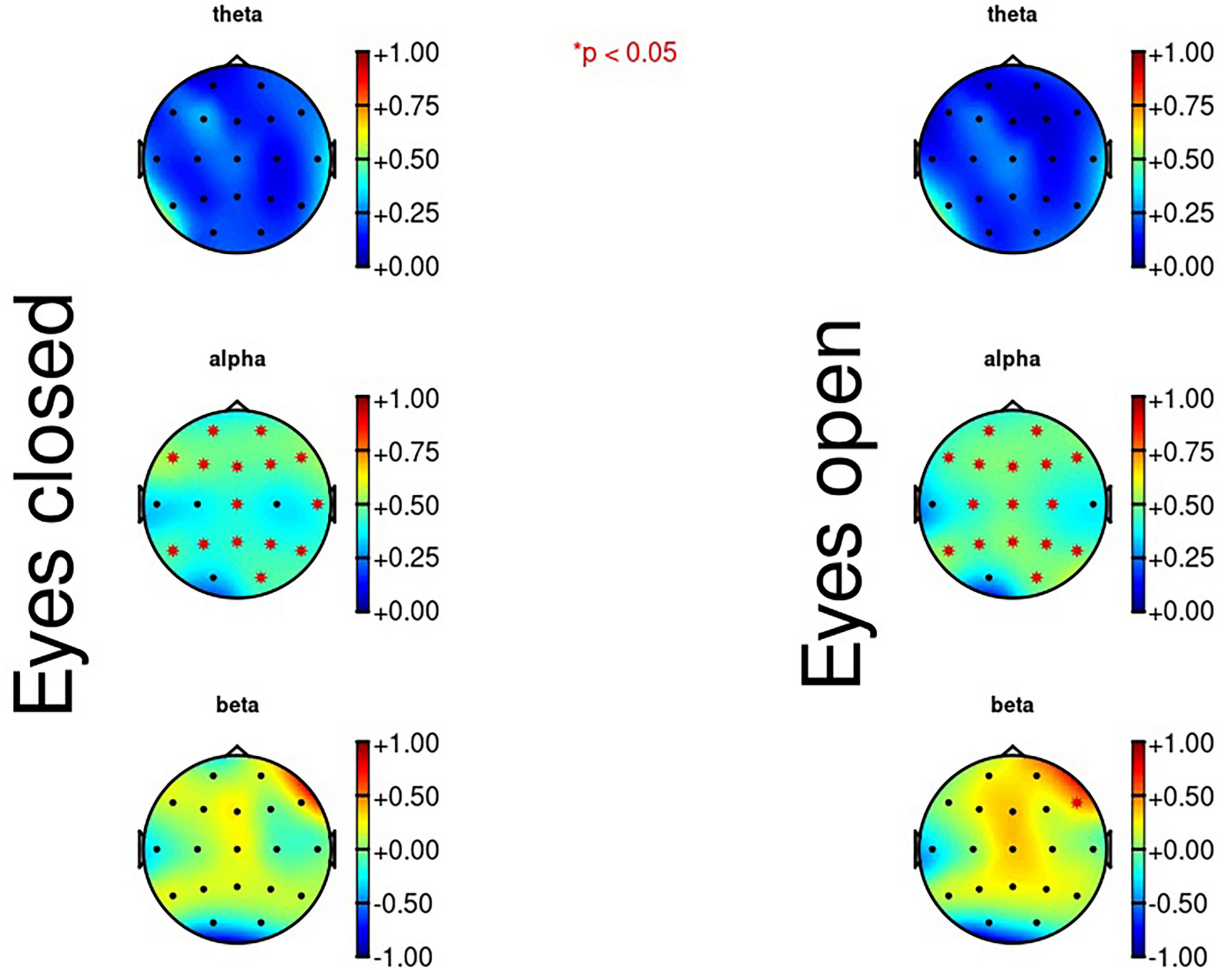
Correlation maps for the EEG resting State and the subscale “women are unknowable”.

Results of the correlations between the QITSO, its subscales and the resting state EEG data are represented in Table 6. The subscale women are unknowable correlated significantly with three of the resting state EEG measures, beta waves during eyes closed, alpha waves during eyes open, and beta waves during eyes open. These results are summarized in Figure 7. The difference score for the theta waves comparing eyes open to eyes closed correlated significantly with the subscales dangerous world and women are unknowable. The difference score for the alpha waves correlated significantly with the subscale children as sexual beings.

**Table 6:** Correlations between the QITSO and the resting state EEG data.

Resting state EEG variables	Total score QITSO	Children as sexual beings	Entitlement	Dangerous world	Uncontrollability	Women are unknowable	Women as sexual objects
Theta.EC	0.162	0.57	˗0.119	˗0.296	0.000	0.008	0.069
Alpha.EC	0.236	0.088	˗0.032	0.269	0.109	0.350	0.122
Beta.EC	0.215	0.054	0.094	0.272	0.046	0.394^*^	0.133
Theta.EC	0.199	0.006	˗0.071	0.346	0.068	0.66	0.116
Alpha.EC	0.343	0.090	0.016	0.324	0.205	0.463^*^	0.266
Beta.EC	0.309	0.079	0.039	0.328	0.072	0.515^**^	0.259
Theta difference	0.197	˗0.269	0.006	0.503^*^	0.042	0.538^*^	0.370
Alpha difference	˗0.172	˗0.582^*^	0.140	0.103	˗0.037	0.407	0.189
Beta difference	˗0.332	˗0.158	0.211	˗0.314	˗0.289	0.018	˗0.260

** *p* < 0.01;

* *p* < 0.05.

### QITSO and the ECG resting state

Results of the correlations between the QITSO, its subscales, and the resting state ECG data are represented in Table 7. As can be seen, the subscale entitlement correlated significantly with three measures, namely power in the low-frequency band during eyes closed power in the high-frequency band during eyes closed and the power in the low-frequency band during eyes open.

**Table 7:** Correlations between the experimental QITSO and the resting state heart rate data.

Resting state heart rate variables	Total score QISO	Children as sexual beings	Entitlement	Dangerous world	Uncontrollability	Nature of harm	Women are unknowable	Women as sexual objects
MeanRR.E C	˗0.041	˗0.12	0.047	˗0.100	˗0.001	˗0.116	0.141	0.070
MeanRR.E C	0.032	0.026	˗0.077	0.056	0.024	0.089	˗0.110	0.001
SDNN.EC	0.018	˗0.083	0.337	0.018	0.044	0.084	˗0.084	0.044
RMSDD.EC	0.061	˗0.063	0.312	˗0.029	0.150	0.121	˗0.123	0.116
VLF.EC	˗0.018	˗0.068	0.161	0.047	0.028	0.041	0.031	˗0.060
LF.EC	˗0.042	˗0.205	0.484^*^	0.033	˗0.059	0.011	˗0.084	0.092
HF.EC	0.041	˗0.134	0.389^*^	0.063	˗0.080	0.044	˗0.071	0.073
LFHF.EC	˗0.143	˗0.059	0.004	˗0.069	˗0.244	˗0.068	0.006	0.002
MeanRR.E O	˗0.004	0.067	0.073	˗0.112	˗0.024	˗0.005	0.029	0.007
MeanHR.E O	0.007	˗0.038	˗0.087	0.068	0.068	0.048	˗0.001	˗0.022
SDNN.EO	˗0.147	˗0.141	0.204	˗0.111	˗0.194	0.047	˗0.083	˗0.044
RMSDD.E O	0.035	˗0.091	0.273	˗0.007	0.052	0.201	˗0.114	0.055
VLF.EO	˗0.241	˗0.013	0.089	˗0.250	˗0.367	˗0.045	˗0.114	˗0.119
LF.EO	˗0.100	˗0.305	0.394^*^	0.049	˗0.096	0.009	˗0.066	0.061
HF.EO	0.013	˗0.123	0.354	0.025	0.003	0.142	˗0.097	0.056
LFHF.EO	˗0.157	˗0.205	˗0.068	0.024	˗0.135	˗0.229	0.075	˗0.013

* *p* < 0.05.

## Discussion

The main goal of this exploratory study was to see whether a psychophysiological profile could be established in a community sample that underlies the self-report measurement of seven implicit beliefs found in sexual offenders as measured by the QITSO. These seven beliefs are children as sexual objects, entitlement, dangerous world, uncontrollability, nature of harm, women are unknowable, and women are sexual objects. The scores on the QITSO in this study were on the lower end, which was expected since the study was conducted in a community sample.

First, task performance for both the Go/NoGo task and the auditory oddball task was found to be consistent with the literature, so the task manipulations were adequate. Correlations between the QITSO and its subscales and the task performance items for both tasks showed no significant correlations. For the EEG data, the electrode best displaying the Go/NoGo waveform was the Fz electrode, and for the auditory oddball waveform, this was the Pz electrode, which is consistent with the literature. Differences scores between the two conditions were calculated for these two electrodes. The difference scores for both Go/NoGo and the auditory oddball task were then correlated with the QITSO and its subscales. Results showed a significant, positive correlation between the auditory oddball difference score and the subscale children as sexual beings. Bernat et al. (2007) found that violent offenses, but not non-violent offenses, were related to a reduced P300 amplitude, as investigated in a sample of 138 male residents in a medium security correctional facility. According to the literature, a reduced P300 amplitude might be indicative of a deficiency in the effective deployment of resources to cognitively evaluate task-relevant information, which might increase the risk of aggression (Bernat et al., 2007). P300 has been identified as an indicator of neurobiological vulnerability, underlying externalizing disorders such as substance dependence, conduct disorder and antisocial personality disorder (Joyal et al., 2007).

As discussed previously, a study conducted by Steele et al. (2013) investigated the P300 in relation to pleasant-sexual, unpleasant, and neutral stimuli in a normal population. The results of this study indicated that P300 mean amplitude in the neutral condition was less positive compared to both the unpleasant and pleasant-sexual conditions. Also, the P300 mean amplitude for the pleasant-sexual condition was more positive than that the unpleasant and neutral conditions. The difference between the study by Steele et al. (2013) and the current study is the fact that the study of Steele and colleagues used sexually related stimuli and compared this to neutral stimuli. The current study only used neutral stimuli and therefore assessed a more structural, underlying difference in P300 amplitude, which is not dependent upon the type of stimuli used. Looking for a more structural difference in P300 amplitude has been done in relation to externalizing disorders, by using neutral stimuli. However, this kind of research has not been done before with regard to sexual offences. Therefore, it is not possible to draw definite conclusions about the relation between the P300 amplitude and the QITSO and its subscales. The current study is the first to investigate biomarkers of sexually deviant behavior and indicates that there might be an underlying difference in P300 amplitude in relation to the scores on the QITSO. However, future research in a clinical sample of sex offenders should explore this relationship further to draw more definite conclusions.

The last step was to correlate the various physiological variables from the resting state block with the QITSO and its subscales. Results showed that the subscale women are unknowable correlated significantly with three resting state variables, namely beta wave during eyes closed, alpha wave during eyes open, and beta wave during eyes open. Furthermore, the subscale entitlement correlated significantly with three resting state variables, namely power in the low-frequency band during eyes closed, power in the high-frequency band during eyes closed, and power in the low-frequency band during eyes open. In the current study, theta waves were mainly measured frontally, alpha waves mainly parietal, while the beta power has no clear skull distribution. This is consistent with what is generally found in the literature (Greer et al., 2021).

Regarding alpha waves, it was found that there was a positive correlation with the subscale women are unknowable. Lower alpha waves could be interpreted as a sign of arousal or brain activity, since alpha waves indicate the degree of cortical activation. Activated brain areas show lower levels of alpha waves in relation to inactive brain areas (Rezaei et al., 2019). In a study conducted by Zuvok et al. (2008), it was assessed whether there are specific EEG abnormalities in four groups of subjects; offenders of violent criminal activity evaluated as impulsive; individuals who committed no criminal activity; violently deliberately behaving delinquents; delinquents performing property criminal activities. The results of this study showed that only in the group of impulsive criminals, significant EEG abnormalities were found. This group of criminals also showed significantly more alpha abnormalities compared to the other three groups of subjects. Another study conducted by Ortega-Noriega et al. (2015) investigated the case of a psychopath prisoner evaluated through QEEG. Results showed an alpha decrease in the subject and the occipital alpha medium frequency was below average for the subject’s age. However, in relation to sexual offending, there has not been any research on alpha waves and alpha activity in the brain.

Regarding beta brainwaves, they are found to be associated with motor domains, such as contracting muscles and they are also related to somatosensory domains (Baker, 2007). Increased beta activity has been found to be associated with schizophrenia (Uhlhaas, 2013), with anxiety (Roohi-Azizi et al., 2017), and in patients with PTSD (Roohi-Azizi et al., 2017), whereas decreased beta rhythms are found in the EEG of patients diagnosed with obsessive–compulsive disorder (OCD) (Karadag et al., 2003), patients with ADHD (Karadag et al., 2003), patients with autism (Cowan and Markham, 1994), and people with an addiction (Rangaswamy et al., 2002). However, there is currently no literature on the relation between beta brainwaves and sexual offending.

Regarding the resting state ECG data, the subscale entitlement correlated significantly with power in the low-frequency band during eyes closed, power in the low-frequency band during eyes open, and power in the high-frequency band during eyes closed. The bands reflect the activity of the autonomic nervous system and heart rate variability is influenced by this nervous system. Voggt et al. (2015) conducted a study on heart rate variability in patients with bipolar disorders and found that the low-frequency and high-frequency HRV were significantly reduced in patients with bipolar disorder compared to healthy controls. Patients with panic disorder were also found to have reduced low-frequency power compared with the control group (Gorman and Sloan, 2002). Regarding depression, previous research has shown increased values of low-frequency power for patients with depression in comparison to healthy controls (Hartmann et al., 2019). This could indicate that people with increased low-frequency power are more alert because the autonomic nervous system is responsible for mobilizing the body’s fight-or-flight response in response to perceived threat. As previously discussed, the low-frequency band reflects the sympathetic activity. This could indicate that people with an increased low-frequency power are more alert because the sympathetic nervous system is responsible for mobilizing the body’s fight-or-flight response in response to perceived threat (McCorry, 2007).

Lastly, results of the current study showed a significant correlation between the subscale entitlement and power in the high-frequency band during eyes closed. The high-frequency band reflects parasympathetic activity, and it responds the heart rate variations caused by the respiratory cycle (Blood et al., 2015). Thus far, previous research has found a correlation between reduced high-frequency power with stress, panic, and worry (Shaffer and Ginsberg, 2017), and anxiety disorder and depression (Berntson and Cacioppo, 2004). The current study, however, found a positive correlation between the high-frequency power and the subscale entitlement. Thus, a higher score on the subscale also means more high-frequency power and vice versa. The correlation between this subscale and the high-frequency power, however, is not very strong.

The current study is of scientific and clinical relevance and offers first insights into a possible psychophysiological profile that can be used to supplement responses on the QITSO. In general, it was found that some of the psychophysiological variables used (P300, alpha waves, beta waves, power in the low-frequency band, and power in the high-frequency band) showed a positive correlation with some of the subscales of the experimental QITSO. The biological measures, self-report measures and behavioral measures, were seen in this study as transdiagnostic, separate, additional measures that together can provide more insight to develop a preliminary profile to understand coherence. However, as this is the first study to examine this and it is tested on healthy participants, more research is needed to establish a more definite psychophysiological profile. A limitation of this preliminary exploratory study involves not correction for multiple testing. As this is the first study to examine a psychophysiological profile as a supplement to self-report data regarding sexual offending, it was decided not to use a correction. Furthermore, the data collection of the eyes open and eyes closed conditions was not randomized, so there could be a potential sequence effect. This study represented a first step in establishing biomarkers of sexually deviant behavior, tested in a community sample with non-sexually oriented stimuli. Variation was thus expected to be small; however, this study provides a starting point for future research to investigate biomarkers of sexually deviant behavior in a clinical sample and with both non-sexually oriented and sexually oriented stimuli. Additionally, recruiting a group of female participants in future research can be helpful to differentiate the correlations between self-report measures and the neurophysiological data. When such biomarkers or a psychophysiological profile can be established, it can help counterbalance the limitations in self-report measurements, to get a more objective image of what goes on in the mind of body and sexual offenders.

## Data availability statement

The raw data supporting the conclusions of this article will be made available by the authors, without undue reservation.

## Ethics statement

The Ethics Review Board of the Tilburg School of Social and Behavioral Sciences has approved this study under the overarching study Conscious and unconscious information processing EC-2016.48, 9-12-2016. The patients/participants provided their written informed consent to participate in this study.

## Author contributions

RL, GB, and SB: conceptualization, methodology, validation, and writing – review and editing. RL and GB: software, formal analysis, and data curation. RL, SB, and GB: investigation. RL: resources, visualization, and project administration. RL, GB, SB, and EM: writing – original draft preparation. GB and SB: supervision. All authors contributed to the article and approved the submitted version.

## Conflict of interest

The authors declare that the research was conducted in the absence of any commercial or financial relationships that could be construed as a potential conflict of interest.

## Publisher’s note

All claims expressed in this article are solely those of the authors and do not necessarily represent those of their affiliated organizations, or those of the publisher, the editors and the reviewers. Any product that may be evaluated in this article, or claim that may be made by its manufacturer, is not guaranteed or endorsed by the publisher.
